# Smooth leaflets with curved belly and attachment edge profiles promote adaptive remodeling in tissue-engineered heart valves: an in silico study

**DOI:** 10.1007/s10237-025-01937-8

**Published:** 2025-04-04

**Authors:** Valery L. Visser, Sarah E. Motta, Simon P. Hoerstrup, Frank P. T. Baaijens, Sandra Loerakker, Maximilian Y. Emmert

**Affiliations:** 1 Institute for Regenerative Medicine, University of Zürich, Zurich, Switzerland; 2 Wyss Zürich, University and ETH Zürich, Zurich, Switzerland; 3 Department of Biomedical Engineering, Eindhoven University of Technology, Eindhoven, The Netherlands; 4 Institute for Complex Molecular Systems, Eindhoven University of Technology, Eindhoven, The Netherlands; 5 Charité Universitätsmedizin Berlin, Berlin, Germany; 6 Department of Cardiothoracic and Vascular Surgery, Deutsches Herzzentrum der Charite (DHZC), Berlin, Germany

**Keywords:** Heart valve tissue engineering, Tissue-engineered heart valve, Computational modeling, Collagen remodeling, Biomechanics, Heart valve design

## Abstract

Tissue-engineered heart valves (TEHVs) are promising valve replacements due to their potential to regenerate into living heart valves, capable of growth and adaptation. Previous TEHVs showed promising results, but often developed progressive leaflet retraction in the long term. In a prior proof-of-concept study, we demonstrated that a novel geometry with more native-like mechanical behavior could give rise to more adaptive remodeling, thereby minimizing leaflet retraction in vivo. In the current study, we aimed to systematically analyze the impact of TEHV geometry on in vivo remodeling under both pulmonary and aortic conditions. Using a bio-inspired in silico framework, we predicted cell-driven, mechano-mediated remodeling in TEHVs post-implantation. Two parameterized valve designs were evaluated under both pulmonary and aortic pressure conditions. The results indicate that a valve design with smooth leaflets, a curved belly profile, and medium to wide attachment edge effectively minimizes stress concentrations and reduces the risk of valve insufficiency in both conditions. Additionally, this design should be tailored to specific hemodynamic conditions to prevent retraction in pulmonary applications and excessive stress concentrations in aortic applications. These insights provide essential guidelines for optimizing TEHV designs, aiming to promote functional remodeling and maintain valve functionality over time, thereby advancing the development of next-generation TEHVs with enhanced long-term outcomes.

**Supplementary Information:**

The online version contains supplementary material available at 10.1007/s10237-025-01937-8.

## Introduction

Cardiovascular diseases are a major burden on today’s society, with heart valve defects alone affecting millions of people worldwide (Martin et al. 2024). Valvular heart disease, frequently marked by a loss of valve functionality, is often alleviated through the replacement of the affected valve with either a mechanical or bioprosthetic valve. However, mechanical valves necessitate lifelong anticoagulant therapy, while bioprosthetic valves are susceptible to structural failure due to progressive calcification (Dvir et al. 2018; Fuller et al. 2021; Jaffer et al. 2015). Importantly, both types of valve replacements do not have regenerative abilities, thereby preventing their growth or repair when needed. This is particularly problematic for pediatric patients who require multiple invasive interventions throughout their lifetime (Fuller et al. 2021).

A promising alternative for the current clinically available prostheses is represented by tissue-engineered heart valves (TEHVs) (Blum et al. 2022; Hjortnaes et al. 2009; Chester and Grande-Allen 2020). Tissue engineering aims to generate implants that gradually transform into living tissues that exhibit native-like functionality and adaptive capacity. In early generations of TEHVs, a biodegradable scaffold was seeded with cells and cultured in vitro to induce the production of extracellular matrix (ECM). The end product was an engineered hybrid valve composed of cellular and tissue components, with residual remnants of scaffold material. However, upon implantation, these TEHVs often showed maladaptive remodeling, marked by chronic inflammation, fibrosis, and leaflet retraction, leading to valve stenosis and insufficiency (Gottlieb et al. 2010; Flanagan et al. 2009; Schmidt et al. 2010).

In an effort to streamline the TE approach logistically, while concurrently mitigating maladaptive cell-mediated remodeling caused by excessive host responses, different methods with acellular materials have been developed to generate living TEHVs *in situ*. These TEHVs elicit an acute inflammatory response upon in vivo implantation, which triggers the recruitment of endogenous cells (Fioretta et al. 2020). Ideally, these cells induce tissue remodeling, upon which the initial inflammatory response is resolved. For this *in situ* approach, biodegradable synthetic scaffolds can be used, offering the advantage of off-the-shelf availability and eliminating the need for in vitro cultures (Capulli et al. 2017; Machaidze et al. 2023; Kluin et al. 2017; Uiterwijk et al. 2020). Still, although promising results have been obtained, controlling the balance between inflammation, scaffold degradation, and tissue production *in vivo* has proven challenging, leading to several events of maladaptive remodeling with leaflet thickening and deterioration of functionality over time (Machaidze et al. 2023; Kluin et al. 2017; Uiterwijk et al. 2020).

Another recent approach is to develop TEHVs of decellularized hybrid tissue-engineered matrices (TEMs), where cells are stimulated to produce extracellular matrix on a biodegradable scaffold in vitro, and constructs are decellularized before in vivo implantation. Upon recellularization in situ, this approach showed improved short-term remodeling in animal studies (Driessen-Mol et al. 2014; Lintas et al. 2018; Motta et al. 2018; Reimer et al. 2017). Nevertheless, long-term remodeling led to the progressive development of leaflet retraction, resulting in the deterioration of valve functionality (Driessen-Mol et al. 2014; Reimer et al. 2017).

A substantial improvement was achieved in recent studies where it was shown that the TEHV geometry at implantation could favorably steer the remodeling of decellularized TEM-based TEHVs. Since it was demonstrated previously that the geometry of the TEHVs affects the mechanical state of the TEVHs, and multiple studies have shown that cells respond to mechanical stimuli, for example, by adjusting their proliferation rate, orientation, and ECM production (Seliktar et al. 2003; Garoffolo and Pesce 2019), it was hypothesized that TEHV geometries with more native-like deformation patterns could substantially improve TEHV remodeling. Indeed, an in vivo evaluation of TEHVs with a computational modeling-inspired valve geometry demonstrated that these TEM-based TEHVs maintained functionality and pliable leaflets during the complete 1-year follow-up period when implanted in the pulmonary position in sheep (Loerakker et al. 2013; Emmert et al. 2018; Motta et al. 2020).

This first proof-of-concept study revealed the importance of geometry on TEHV remodeling and functionality in the pulmonary position. Nonetheless, it is still unknown which geometrical features are essential in guiding the remodeling response toward functional adaptation. Furthermore, the effect of valve geometry on TEHV remodeling under substantially higher aortic pressure conditions remains unclear.

Therefore, in the current study, we employed a previously developed bio-inspired computational framework, which has previously already been used to analyze cell-mediated remodeling in TEM-based TEHVs in vivo (Loerakker et al. 2016; Emmert et al. 2018) to address these questions. The framework integrates TEHV mechanics and cell-mediated tissue remodeling in collagenous tissues (Obbink-Huizer et al. 2014; Loerakker et al. 2016). With this framework, we systematically investigated how geometrical design impacts mechano-mediated remodeling in TEHVs, and its consequences for maintaining TEHV functionality, in both pulmonary and aortic pressure conditions.

## Methods

### Parameterized geometries

**Fig. 1:**
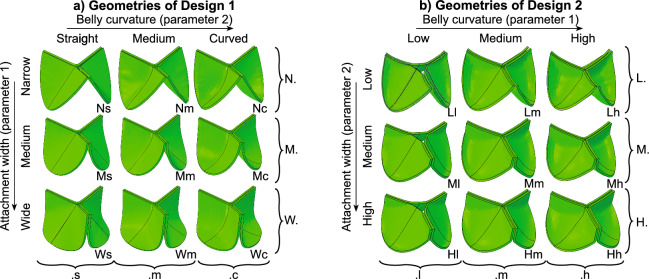
TEHV design variations, with their two-letter nomenclature, based on the attachment shape and belly curvature as well as one-letter nomenclature for each group of three valve geometries with a similar design feature. Obtained valve geometries for Design 1 (**a**) and Design 2 (**b**)

**Fig. 2:**
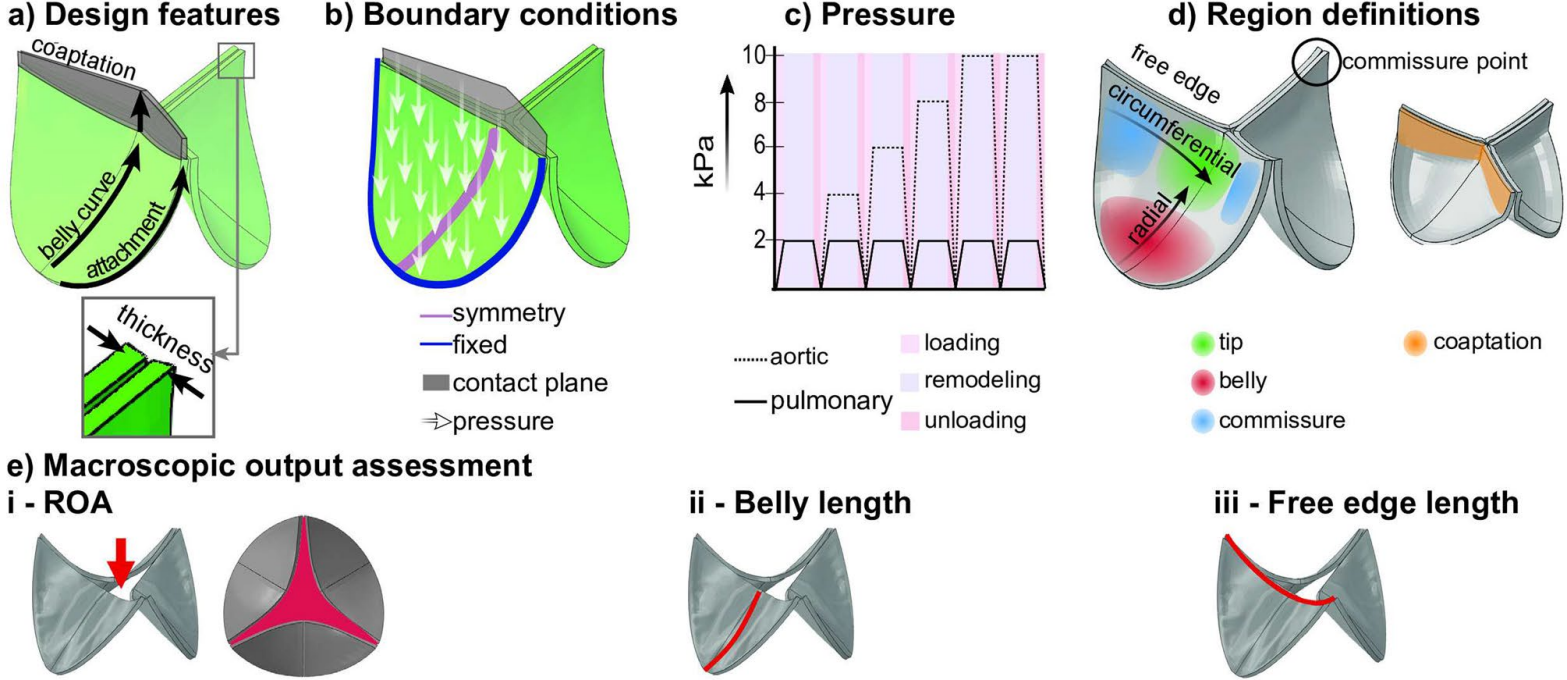
Design features used to generate geometrical variations (**a**), boundary conditions in simulations (**b**), profile of pressure on the leaflets (**c**), definition of regions and orientations in the valves (**d**), and depiction of measures used for macroscopic evaluation of valves after remodeling (**e**)

In the current study, two parameterized designs were used, each capable of generating various valve geometries to systematically analyze how design features affect remodeling in TEHVs. Generally, valve designs with a radius (*R*) of 11.5 mm and a maximum valve height (*H*) of 14.3 mm were simulated. Importantly, the variations within both designs concerned the curvature of the leaflet belly and the attachment edge (Fig. 2a).

#### Design 1

Design 1 was based on Gulbulak et al. (2020) and has two curves that generate the attachment edge (attaching the leaflet to the stent/annulus) and a line defining the belly curvature of the leaflet.

The first curve is shaped by a cubic Bezier curve with four points ($P_0$-$P_3$). For a half leaflet, two points define the start and endpoint ($P_0$ and $P_3$). These are defined as follows: $P_{0_y} = 0$, $P_{0_z} = 0$, $P_{3_y} = 14.3$ (valve height *H*), and $P_{3_z} = -(2 \cdot \frac{\pi }{6}) \cdot R$ (using radius *R*). One of the coordinates of each point is always fixed: $P_{1_y}=0$ and $P_{2_z}=P_{3_z}$. A single parameter ($D_{1,1}$) controls the curvature by shifting both $P_1$ and $P_2$, defined as $P_{1_z} = D_{1,1} \cdot P_{3_z}$ and $P_{2_y} = D_{1,1} \cdot P_{3_y}$. This curve is subsequently wrapped around a cylinder to create the three-dimensional curve.

The second curve describes the curvature of the belly and is guided by three points ($P_4$ through $P_6$. $P_4$ is a fixed control point with $P_{4_x}=-R$, $P_{4_y}=0$. $P_6$ is another fixed point. Since the leaflet’s free edge has an angle of 17 degrees from the commissure point, the *y*-coordinate of the leaflet tip is calculated as follows: $P_{6_y}=P_{3_x}-|\sin (\frac{17}{180})|\cdot (\frac{2\cdot \pi \cdot R}{3}/2)$, $P_{6_x} = 0.125$ was empirically determined in Gulbulak et al. (2020). Geometrical variations in the belly curve are determined by adjusting the guiding point $P_5$. This point is fixed in the *x*-direction ($P_{5_x} = 0$) but can move in the *y*-direction, controlled by the parameter $D_{1,2}$.

Subsequently, the leaflet is generated by horizontally connecting 26 points along the attachment spline to 26 corresponding points on the belly spline. A 2 mm fillet is added around the belly spline to smooth the connections. A surface is fitted through these curves, and, finally, a mesh is extruded from this surface. A complete table with the curves’ coordinates and a graphical illustration of the defining curves are added in Appendix 1.

#### Design 2

Design 2 was based on Hamid et al. (1986), similar to Loerakker et al. (2013). The leaflet is described as an elliptic paraboloid $\frac{x^2}{{D_{2,1}}^2}+\frac{y^2}{{D_{2,2}}^2}-z=0$ rotated by angle $\tan ^{-1}(\frac{R}{2H})$ around the *x*-axis. Points that cross into the domain of other leaflets are displaced to the plane $y=\tan (30\deg )||x||$ to create the initial coaptation area. To finish the geometry, all points outside the valve radius are removed. Here, $D_{2,1}$ defines the circumferential curvature ($2.8\le D_{2,1}\le 4.0$) and $D_{2,2}$ defining the radial curvature of the leaflet geometry ($1.4\le D_{2,2}\le 2.4$).

#### Design variations

For each parametrized geometry, three values for each parameter were assessed, resulting in nine unique geometries for each design (Fig. 1a, b). For Design 1, the full range of values for the guiding points of the Bézier curves were used. Design 2 parameters were restricted to ensure that variations initially yielded fully closed valve geometries. Groups sharing similar features, such as valves with wide commissures in Design 1, were assigned similar codes (e.g., “.W”), as shown in Fig. 1a, b. This resulted in a two-letter code for each design, reflecting the curvatures of both the belly and attachment edges (Fig. 1a, b).

The resulting geometry variations exhibit both shared and distinct properties. The directly defined curvatures in the central region and attachment edge are affected by the parameter variations, but consequently also the (initial) contact area between adjacent leaflets (i.e., coaptation) varies between geometries. In addition to this, Design 1 displays a more smooth leaflet profile compared to Design 2.

All designs were additionally evaluated with increased coaptation, by extending the leaflet tip in Design 1 and increasing valve height in Design 2 (to H=16.31 mm). Furthermore, different thicknesses (0.4 mm, 0.6 mm, and 0.8 mm) were tested (Fig. 2a). Due to symmetry, only half a leaflet was modeled and meshed with brick elements with quadratic interpolation (C3D20 elements), with two elements in the thickness direction and a total of 346 elements on average.

### Material model

A previously developed material model was utilized to describe the tissue’s mechanical behavior and mechano-mediated remodeling, and implemented in a user subroutine (UMAT) in Abaqus Standard (Abaqus 2014, Dassault Systèmes Simulia Corp. Johnston, RI, USA). In this section, a brief explanation of the model’s concepts is provided. More elaborate descriptions can be found in Appendix 2 and in previously published papers (Obbink-Huizer et al. 2014; Ristori et al. 2016; Loerakker et al. 2016).

The model includes three main tissue constituents: stress fibers of fibroblast-like cells, collagen fibers, and an isotropic component representing the non-fibrous ECM. The total Cauchy stress equals the sum of the stresses in the stress fibers ($\varvec{\sigma }_{sf}$), collagen fibers ($\varvec{\sigma }_{cf}$), and the non-fibrous ECM ($\varvec{\sigma }_{ecm}$):

1 $$\begin{aligned} \varvec{\sigma }=\varvec{\sigma }_{sf}+ \varvec{\sigma }_{cf}+\varvec{\sigma }_{ecm}. \end{aligned}$$

The anisotropic contributions of the stress fibers and collagen fibers are modeled using 30 equally spaced fiber directions in the plane of the leaflet, spanned by two orthogonal vectors $\vec {v}_1$ and $\vec {v}_2$ in the circumferential and radial direction, respectively. The vector $\vec {e}_{f0}^i$ denotes fiber direction *i* in the reference configuration, and $\vec {e}_{f}^i$ is the corresponding fiber direction in the current, deformed configuration.

#### Cellular stress fibers

Fibroblast-like cells are included in the model since experimental studies indicate that cellular traction forces are critical for collagen compaction (Meshel et al. 2005; Shi and Vesely 2003; Feng et al. 2003). Computational models have predicted this effect to be dominant in the development of valve insufficiency in TEM-based TEHV remodeling under low hemodynamic loads (like pulmonary pressure conditions) (Loerakker et al. 2016). This was identified as the main mode of failure in previous generations of TEHVS (Driessen-Mol et al. 2014). Therefore, cellular traction forces are included through a stress fiber model that describes their role in compacting ECM via actin polymerization into stress fibers.

The total actin volume fraction ($\phi _a$) is, therefore, described as the sum of free monomers ($\varphi _{mon}$) and monomers polymerized into stress fibers ($\varphi _{sf}^i)$ in *N* directions:

2 $$\begin{aligned} \phi _a=\varphi _\textrm{mon}+\frac{1}{N}\sum _{i=1}^N \varphi _{sf}^i. \end{aligned}$$

For each stress fiber volume fraction, the stress in direction *i* is dependent on the global Green Lagrange strain ($\epsilon ^i$) and strain rate ($\dot{\epsilon }^i$):

3 $$\sigma _{{sf}}^{i} = \sigma _{{\max }} \left( {f_{{\varepsilon ,a}} \left( {\varepsilon ^{i} } \right) + f_{{\varepsilon ,p}} \left( {\varepsilon ^{i} } \right)} \right)f_{{\dot{\varepsilon }}} \left( {\dot{\varepsilon }^{i} } \right),$$

with $\sigma _{max}$ the maximum stress fiber stress, the Green Lagrange strain $\epsilon ^i=\frac{1}{2}((\lambda ^i_f)^2-1)$, and the global fiber stretch $\lambda ^i_f=\sqrt{\vec {e}^i_{f0}\cdot \varvec{F}^T \cdot \varvec{F} \cdot \vec {e}^i_{f0}}$, with $\varvec{F}$ the deformation gradient tensor.

The relation between stress and strain is dependent on active actomyosin contraction $(f_{\epsilon ,a}(\epsilon ^i))$, passive strain hardening $(f_{\epsilon ,p}(\epsilon ^i))$, and the effect of strain rate $(f_{\dot{\epsilon }}({\dot{\epsilon }}^i))$, as described in Appendix 2.

The reorientation of the cells is resembled by the remodeling of the stress fibers, which is, in turn, described by the evolutions of the volume fractions of polymerized actin monomers in the different fiber directions:

4 $$\begin{aligned} \frac{ {\text{d}} \varphi ^i_{sf}} {\text{dt}} =(k_0^{sf}+k_1^{sf}\sigma _{max}f_{e,a}f_{\dot{\epsilon }}) \varphi _{mon}-k_d^{sf}\varphi _{sf}^i. \end{aligned}$$

Here, the parameters $k_0^\textrm{sf}$ and $k_d^\textrm{sf}$ describe the turnover of stress fibers by polymerization and depolymerization, respectively, that is not mechano-mediated. On the other hand, parameter $k_1^\textrm{sf}$ scales the mechano-regulated stress fiber polymerization. The total stress exerted by the stress fibers is the sum of the stresses in each fiber direction:

5 $$\begin{aligned} \varvec{\sigma }_{sf}=\frac{1}{N}\sum _{i=1}^N \varphi _\textrm{sf}^i\sigma ^i_{sf}\vec {e}^i_f\vec {e}^i_f. \end{aligned}$$

As the strain rate is determined by both the loaded and unloaded configurations, the cyclic behavior of loading and unloading of the valve had to be considered. To prevent excessive computational costs, not the complete load history is simulated but an analytical approximation is used to determine the evolution of the stress fiber volume fractions due to dynamic loading conditions: (Ristori et al. 2016):

6 $$\frac{{{\text{d}}\varphi _{{{\text{sf}}}}^{i} }}{{{\text{dt}}}} = \frac{1}{{\tau _{{{\text{sf}}}} }}\left( {\varphi _{{{\text{sf}},p}}^{i} - \varphi _{{{\text{sf}}}}^{i} } \right),$$

where the fiber volume fraction in direction *i* evolves toward the approximated equilibrium given the dynamic mechanical conditions, which is represented by the preferred fiber volume fraction $\varphi ^i_{sf,p}$. Here, time constant $\tau _{sf}$ determines the remodeling speed of the stress fibers. The derivation of $\varphi ^i_{sf,p}$ is described in Appendix 2, and for more elaborate derivations, the reader is referred to Ristori et al. (2016).

#### Collagen fibers

The model incorporates cell-mediated collagen compaction via a multiplicative split of the collagen fiber stretch in direction *i* in an elastic ($\lambda _\textrm{cf,e}$) and a (negative) growth part ($\lambda _{cf,g}$):

7 $$\begin{aligned} \lambda ^i_\textrm{cf}=\lambda ^i _\textrm{cf,e} \lambda ^i_\textrm{cf,g}. \end{aligned}$$

The collagen fibers are assumed to only exert stress in extension ($\lambda _{cf,e}^i>1$) according to:

8 $$\begin{aligned} \sigma _{cf}^i=k^\textrm{cf}_1(\lambda _\textrm{cf,e}^i)^2\left( e^{k^\textrm{cf}_2((\lambda _\textrm{cf,e}^i)^2-1)}-1\right) \text { if } \lambda _\textrm{cf,e}^i>1 \end{aligned}$$

Here $k^\textrm{cf}_1$ and $k^\textrm{cf}_2$ are parameters that define the nonlinear stiffness of the material. The total stress of the collagen is the sum of the stresses in all collagen fibers:

9 $$\begin{aligned} \varvec{\sigma }_\textrm{cf}=\sum _{i=1}^N \varphi _{cf}^i \sigma _\textrm{cf}^i\vec {e}^i_f \vec {e}^i_f, \end{aligned}$$

while the sum of fiber volume fractions in each direction ($\varphi _\textrm{cf}^i$) is the total collagen volume fraction $\phi _\textrm{cf}$:

10 $$\begin{aligned} \sum _{i=1}^N\varphi _\textrm{cf}^i=\phi _\textrm{cf}. \end{aligned}$$

Collagen fibers are compacted by the cells, until a balance between stress fiber and collagen stress is achieved ($\sigma _\textrm{cf}^i=\sigma _\textrm{sf}^i$), thereby assuming that cells cannot contract the collagen fibers when collagen stress exceeds stress fiber stress. The compaction of collagen fibers (in case $\sigma _\textrm{cf}^i < \sigma _\textrm{sf}^i$) evolves toward this stress by determining a preferred growth stretch $\lambda _\textrm{cf,g,p}^i$ that satisfies this stress equilibrium. Subsequently, $\lambda _{cf,g}^i$ evolves, depending on time constant $\tau _\lambda$, according to:

11 $$\begin{aligned} \frac{ {\text{d}} \lambda _{cf,g}^i} {\text{dt}} =\frac{1}{\tau _\lambda }(\lambda _{cf,g,p}^i - \lambda _{cf,g}^i). \end{aligned}$$

Collagen is also continuously turned over, with continuous production and degradation of the fibers:

12 $$\begin{aligned} \frac{ {\text{d}} \varphi _{cf}^i} {\text{dt}} =\frac{ {\text{d}} \varphi _ {cf,prod} ^i} {\text{dt}} +\frac{ {\text{d}} \varphi _{cf,deg}^i} {\text{dt}} , \end{aligned}$$

with a degradation rate $\frac{ {\text{d}} \varphi _{cf,deg}^i} {\text{dt}}$ that decreases with strain, and isotropic collagen deposition $\frac{ {\text{d}} \varphi _{cf,prod}^i} {\text{dt}}$ (see Appendix 2). The deposition rate is constrained by the amount of collagen that was degraded to ensure a constant volume fraction of collagen fibers.

#### Non-fibrous ECM

The isotropic, non-fibrous ECM is modeled as a soft Neo-Hookean material:

13 $$\begin{aligned} \varvec{\sigma} _\textrm{ecm}=\phi _\textrm{ecm}(\kappa _\textrm{ECM}\frac{\ln (J)}{J} {\textbf {I}}+ \frac{G}{J}(\varvec{B}-J^{\frac{2}{3}}\varvec{I})), \end{aligned}$$

with material parameters $\kappa _{ECM}$ the bulk modulus, *G* the shear modulus, *J* the determinant of deformation gradient tensor $\varvec{F}$, and $\varvec{B}=\varvec{F} \cdot \varvec{F}^T$ the left Cauchy-Green tensor.

### Material parameters

**Table 1:** Parameter values used in each simulation

Component	Parameter	Value
Cellular stress fibers	$\phi _a$	0.05 (−)
	$\epsilon _0$	0.12 (−)
	$\epsilon _1$	0.17 kPa
	$\kappa _v$	50 s
	$\sigma _{max}$	40 kPa
	$\kappa _0^{sf}$	$1.5 \cdot 10^{-6}$ $\hbox {s}^{-1}$
	$\kappa _1^{sf}$	0.7 $\hbox {s}^{-1}$ $\hbox {Pa}^{-1}$
	$\kappa _d^{sf}$	$1.0 \cdot 10^{-3}$ $\hbox {s}^{-1}$
	$\tau _{sf}$	5 min
Collagen fibers	$\phi _{cf}$	0.5 (-)
	$k^{cf}_1$	33.01 kPa
	$k^{cf}_2$	11.74 (-)
	$\tau _\lambda$	1 h
	$\tau _{cf}$	12 h
Isotropic ECM	$\phi _{ecm}$	0.45 (-)
	$\nu$	0.3 (-)
	*G*	$1.0 \cdot 10^{-3}$ MPa
	$\kappa _{ECM}$	Estimated through $\frac{2G(1+\nu )}{3(1-2\nu )}$

The material parameters (Table 1) were similar to those used in Emmert et al. (2018). An initial configuration of completely isotropic collagen fibers was adopted (Zaytseva et al. 2023). The stiffness parameters of the collagen fibers ($k^\textrm{cf}_1$ and $k^\textrm{cf}_2$) were additionally varied by 10%. Cell contractility ($\sigma _{max}$) was varied between 20 kPa and 40 kPa to cover the uncertainty in observed cell activation, which defines cell contractility. These values were substantially higher than the values used in Emmert et al. (2018), in order to generate worst-case scenario results and increase the predicted valve-to-valve variability, which allows to better optimize the valve geometry.

Given the possible variability of experimentally determined parameter values, a Taguchi sensitivity analysis was performed to ensure the robustness of our conclusions (see Appendix 3). The analysis, conducted across four geometries (Ns, Nc, Ws, and Wc), revealed quantitative shifts in the results but no changes to the overall conclusions regarding the impact of a design feature on remodeling. This underscores the robustness of our findings regarding the impact of design features on remodeling.

### Boundary conditions

The attachment edge of the valve was constrained in all directions, mimicking the rigid connection of the TEHV to a stent (Fig. 2b). Furthermore, a symmetry boundary condition was applied at the symmetry plane of the leaflet, and a frictionless contact surface was added to model the contact between adjacent leaflets. A pressure of 2 kPa or 10 kPa was imposed on the arterial surface of the valve to mimic pulmonary and aortic diastolic loading, respectively, assuming a period of 0.85 s for loading and unloading.

To improve model convergence, the unsymmetric matrix storage option was utilized using the built-in function in Abaqus Standard. A gradual increase in aortic pressure, from 2 to 10 kPa in 2 kPa increments, was implemented over the first five loading cycles (Fig. 2c). Additionally, a mesh convergence study demonstrated that doubling the element count resulted in less than a 0.1% change in the final ROA after remodeling.

### Output parameters

Valve remodeling and performance were assessed by analyzing global and local (belly, commissure, and tip region) readouts (Fig. 2d) of stress and strain. Macroscopic parameters for leaflet retraction, including the shortening of the leaflet free edge, belly length, and ROA, were assessed (Fig. 2e). Leaflet shortening was identified as a distinct characteristic of the mode of failure of previous generations of TEHVs (Driessen-Mol et al. 2014). Furthermore, ROA, defined as the open area between valve leaflets viewed distally (Fig. 2e–i), is particularly useful because recent techniques enable non-invasive measurement of ROA in vivo, making it a clinically relevant metric (Jeon et al. 2010).

The fiber alignment of both stress and collagen fibers was assessed with an order parameter (Foolen et al. 2014; Ristori et al. 2018):

14 $$\begin{aligned} S=\int \phi _f (\gamma )\cos (2\gamma )d\gamma , \end{aligned}$$

where $\gamma$ represents the angle with respect to the circumferential direction, and $\phi _f(\gamma )$ is the direction-dependent volume fraction of either stress fibers or collagen fibers.

A cost function was used to assess the remodeling and performance of the TEHVs, similar to Szafron et al. (2019). The valves were evaluated based on leaflet stress and diastolic valve closure, measured as the regurgitant orifice area (ROA). High stress concentrations are considered detrimental, as they may damage the leaflet material. Complete valve closure is essential to maintain functionality and prevent regurgitation. A general normalized root square error function for each investigated parameter *P* was used where:

15 $$\begin{aligned} C_P=\left( \frac{T_P-P}{N_P}\right) ^2. \end{aligned}$$

Here $T_P$ is the target value (minimizing stress ($T_{stress}=0$) and valve opening during diastole ($T_{ROA}=0$)), *P* is the value of the parameter of interest, and $N_P$ is the normalization factor (the range between the maximum and minimum value of the respective parameter). The total cost function in case of multiple optimization targets (*n*) was defined as:

16 $$\begin{aligned} C_{tot}=\sqrt{\frac{\sum ^n_{p=1}{(w_p * C_p)}}{\sum ^n_{p=1} w_p}}, \end{aligned}$$

with $w_p$ the weight assigned to each target, allowing prioritization of optimization targets.

## Results

### Smooth leaflets with curved belly profiles minimize stress concentrations both before and after remodeling

**Fig. 3:**
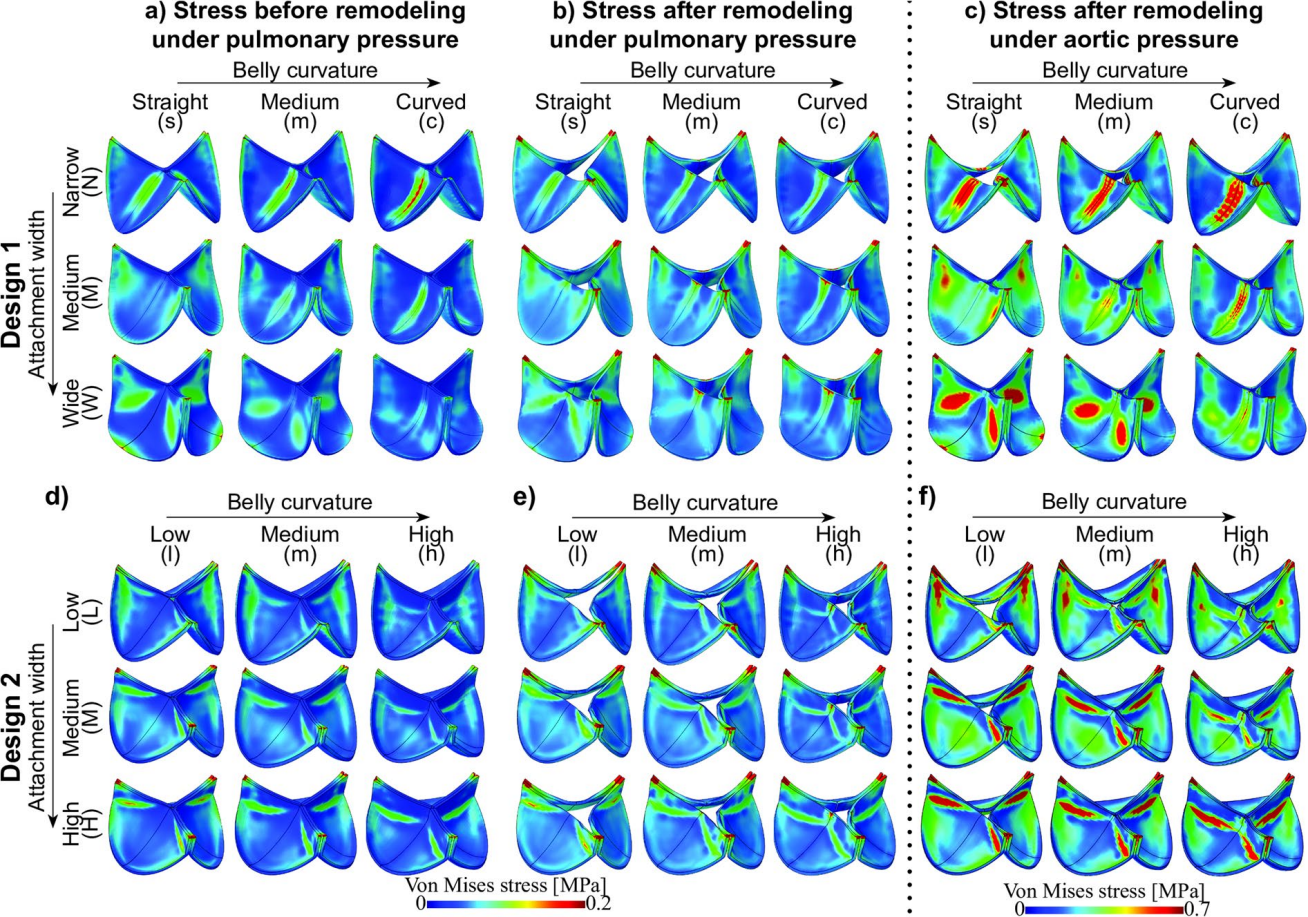
Von Mises stress distributions in TEHVs with different geometries in loaded configuration; under pulmonary pressure before remodeling (**a** and **d**), after remodeling under pulmonary pressure conditions (**b** and** e**), and after remodeling under aortic pressure loading (**c** and **f**). The top row depicts Design 1 (**a**–**c**), and the bottom row depicts Design 2 (**d**– **f**)

Under pulmonary pressure conditions, all the different valve geometries were exposed to relatively low von Mises stresses prior to remodeling (Fig. 3a, d). However, minor stress concentrations were observed at the commissure points in all geometries. Additionally, stress concentrations were notable in the sharp transition regions from the leaflet belly to the coaptation area, especially in all geometries of Design 2.

**Fig. 4:**
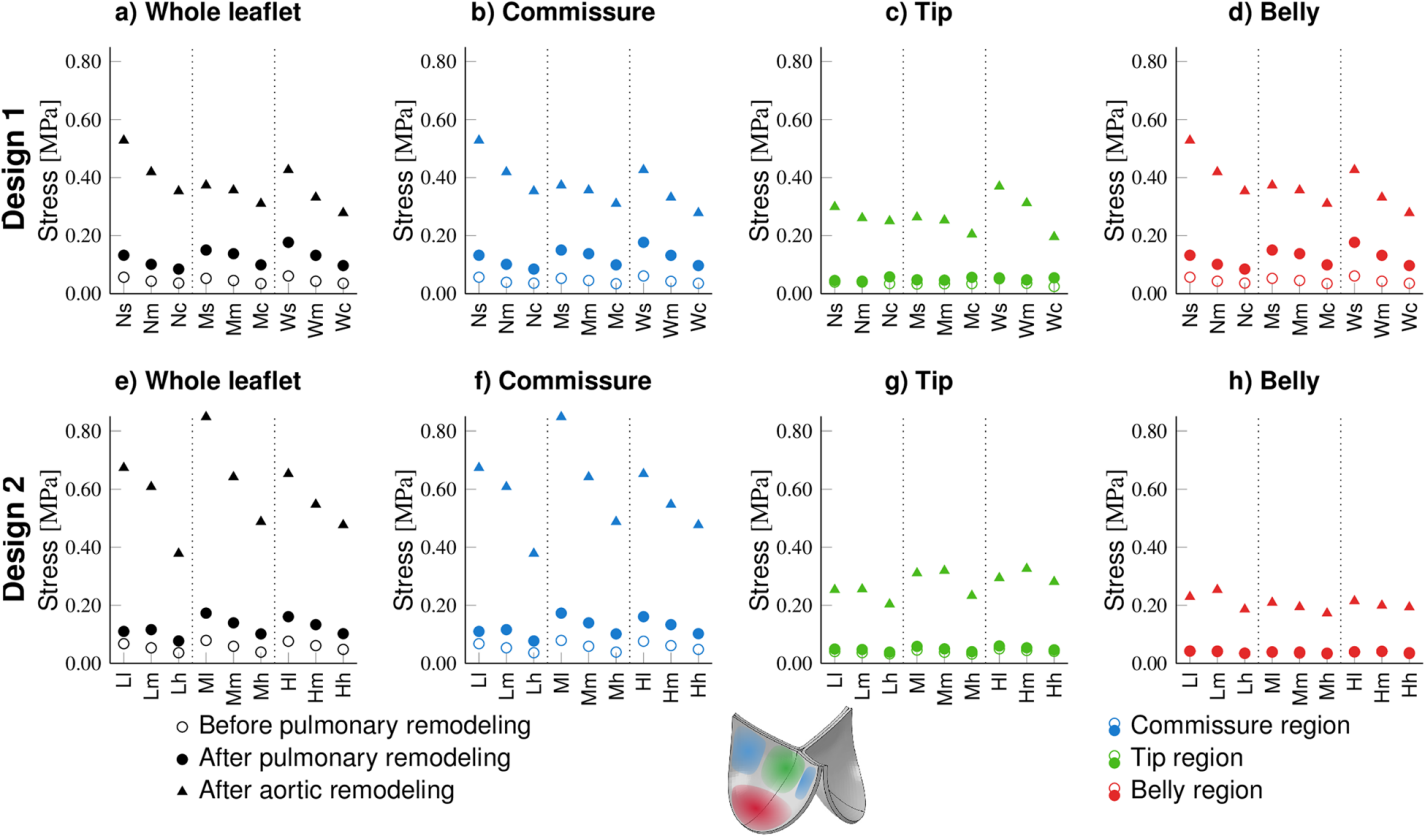
Global and local analysis of the maximal von Mises stress before remodeling under pulmonary conditions and after remodeling under pulmonary and aortic conditions. Peak stress in the whole leaflet (**a** and **e**), the commissure region (**b** and **f**), the tip region (**c** and **f**), and in the belly region (**d** and **h**) are shown, both for Design 1 (**a**–**d**) and Design 2 (**e**–**h**)

These heterogeneities in stress distribution within the leaflets increased after remodeling under pulmonary pressure conditions (Figs. 3b, e and 4), because regions exposed to relatively high stress before remodeling developed a substantial increase in stress post-remodeling. This increase was observed in the commissure regions of all geometries, in the belly regions of geometries of Design 1, in the sharp transition regions of geometries of Design 2, and the more blunt transition regions in some variations of Design 1.

TEHVs remodeling under aortic pressure conditions exhibited substantial higher stress magnitudes and larger stress concentrations in the loaded configuration compared to valves subjected to pulmonary pressures (Figs. 3c, f and 4). Similar to valves remodeling under pulmonary pressure conditions, the stress concentrations after remodeling were primarily located in the sharp transition regions and commissure points.

Importantly, specific design features of valve geometries had a major influence on the stress distribution in TEHVs both before and after remodeling. Specifically, regional analysis (Fig. 4) indicated that geometries with more curved belly profiles (.c and.h) were exposed to substantially smaller stress concentrations, particularly in the commissure regions. In the geometries of Design 1, wider attachment edges (W. and H.) also appeared to slightly decrease stress magnitudes near the commissures. This effect was, however, less pronounced in Design 2.

Notably, in all conditions, the stress concentrations in transition regions and commissure regions in the geometries of Design 1 were considerably lower compared to Design 2 (Fig. 4) which included distinct sharp geometrical transitions from the leaflet belly region to the coaptation area. The smoother geometries of Design 1 did not feature such sharp transition regions, as a result of which stress concentrations in that region were minimal.

### Wide attachment edges and curved belly profiles reduce circumferential strain and increase radial strain before remodeling

**Fig. 5:**
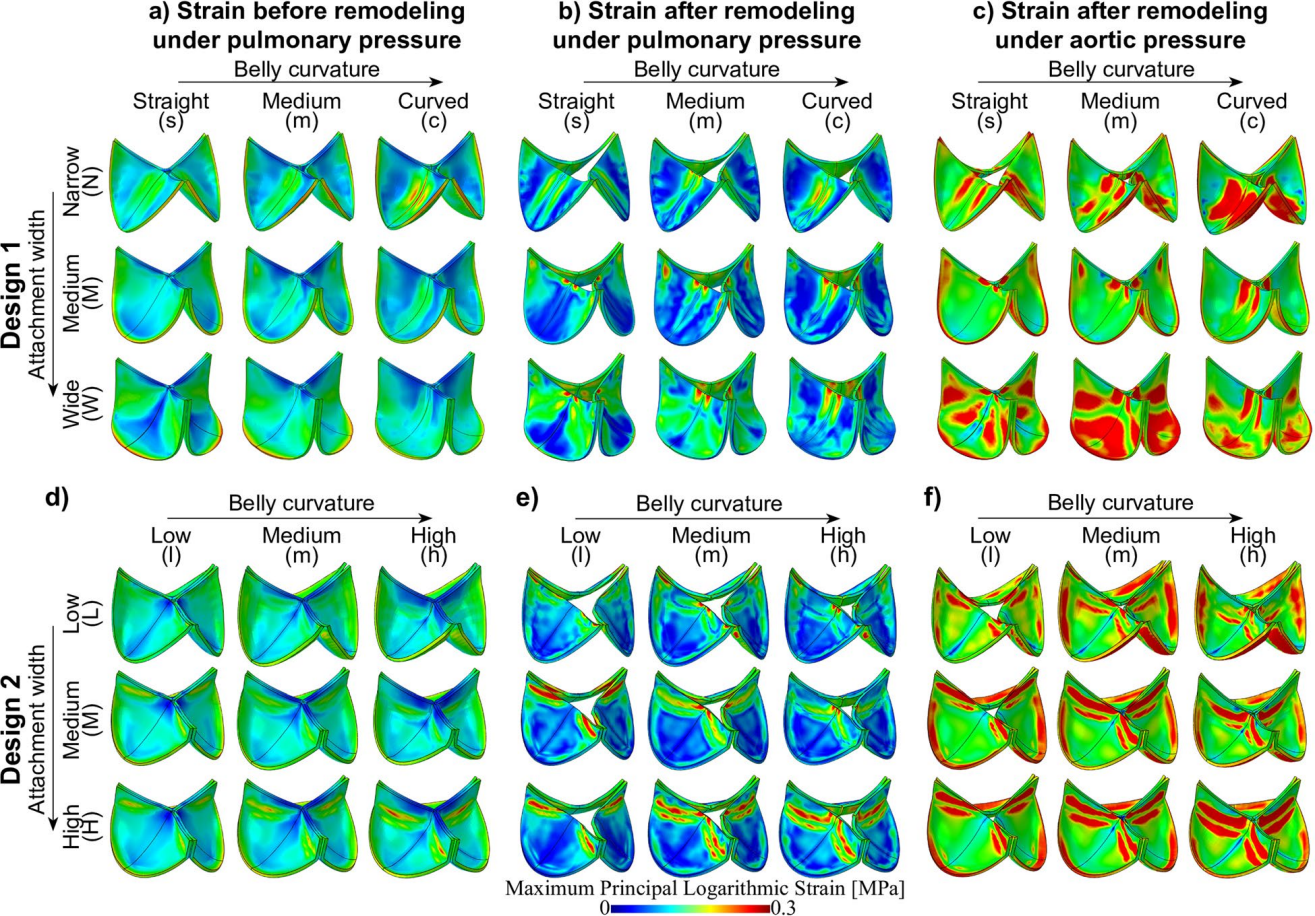
Maximum principal logarithmic strain distributions in TEHVs with different geometries in loaded configuration; under pulmonary pressure before remodeling (**a** and **d**), after remodeling under pulmonary pressure conditions (**b** and **e**), and after remodeling under aortic pressure loading (**c** and **f**). The top row depicts Design 1 (**a**–**c**), and the bottom row depicts Design 2 (**d**–**f**)

After remodeling, variations in collagen orientation result in inhomogeneities in tissue properties. This leads to spatial differences in the stress–strain relationship, requiring independent analyses of stress and strain. The heterogeneity in maximum principal logarithmic strain distributions within the leaflets closely correlated with the stress distributions, with similar variability observed between different valve geometries (Fig. 5). The strain heterogeneity within the leaflets increased due to remodeling, and strain levels were higher under aortic conditions compared to pulmonary conditions, mirroring the phenomena observed in stress distributions.

**Fig. 6:**
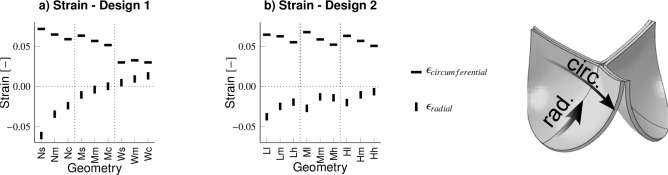
Mean radial and circumferential logarithmic strain before remodeling under pulmonary conditions in the belly region of geometries of Design 1 (**a**) and Design 2 (**b**)

Additionally, directional strain analysis indicated that increased belly profile curvature (.c or.h) and a wider attachment edge (W. or H.) both led to a higher radial strain and lower circumferential strain in the belly region (Fig. 6). For Design 2, the effect of the attachment edge on radial and circumferential strains was less pronounced compared to its effect in Design 1. These findings suggest that leaflet geometry affects the strain anisotropy that serves as a starting point for the remodeling response in TEHVs, where more native-like deformation patterns can be imposed via incorporating sufficient belly curvature and a wide attachment edge.

### Wider attachment edges and curved belly profiles aid in maintaining proper belly profile length and valve functionality during remodeling

**Fig. 7:**
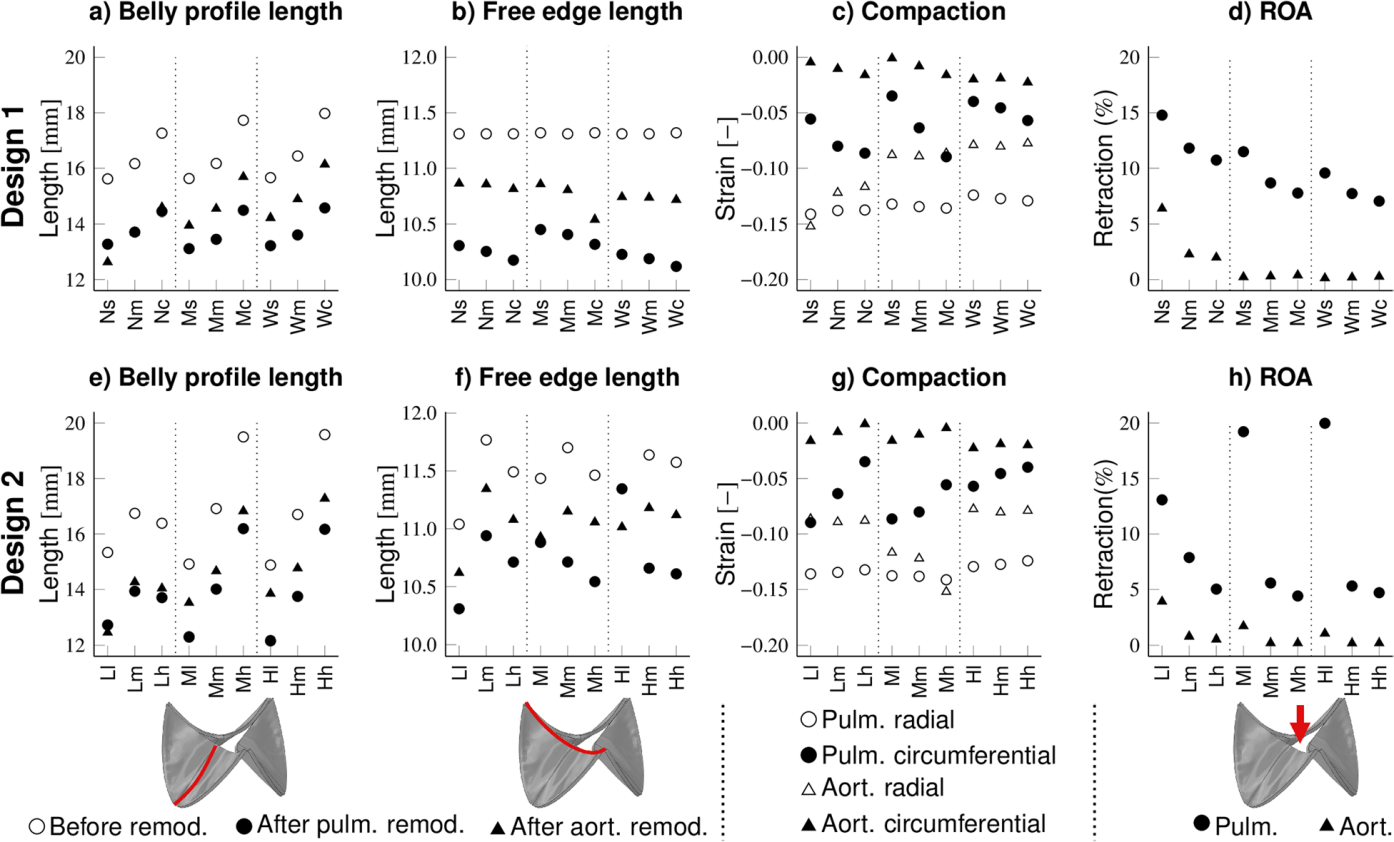
Assessment of leaflet retraction after remodeling under both pulmonary and aortic pressure conditions. The decline in belly profile length (**a** and **e**) or free edge length (**b** and **f**) in the leaflets. The directional compaction in the belly region of the leaflets (**c** and **g**) and the developed valve insufficiency presented as regurgitant orifice area (**d** and **h**)

Geometrical features also influenced the leaflet length before and after remodeling. Specifically, geometries with curved belly profiles (.c or.h), which exhibited larger initial belly profile lengths before remodeling, also resulted in larger belly profile lengths after the remodeling process compared to designs with straighter belly profiles (.s or.l) (Fig. 7a, e). In contrast, the free edge length of all geometries of both designs showed only minor inter-geometry differences, and these differences were largely insensitive to remodeling (Fig. 7b, f).

The reduction of the belly profile profile length was due to compaction in both directions, where a higher degree of radial compaction was observed compared to circumferential compaction (Fig. 7c, g). Interestingly, geometries with more curved belly profiles (.c and.h) and wider attachment edges (W. and H.) exhibited low ROA values after remodeling, even with a certain degree of leaflet retraction (Fig. 7d, h). This indicates that geometries with curved bellies allow more retraction before valvular insufficiency occurs due to the higher initial belly profile lengths.

Aortic pressure conditions prevented the development of valvular insufficiency through two phenomena. First of all, remodeling under aortic pressure conditions led to less compaction compared to remodeling under pulmonary pressure conditions in most geometries (Fig. 7c, g). Secondly, the few designs that obtained similar final belly profile lengths in the unloaded state after remodeling under either pulmonary or aortic conditions (i.e., geometries with narrow attachment edges (N. or L.), Fig. 7a, e) had substantially smaller ROAs under aortic pressure conditions due to the larger pressure-induced deformations in TEHV leaflets in those situations.

### Collagen and stress fiber alignment after remodeling is mildly circumferentially anisotropic

**Fig. 8:**
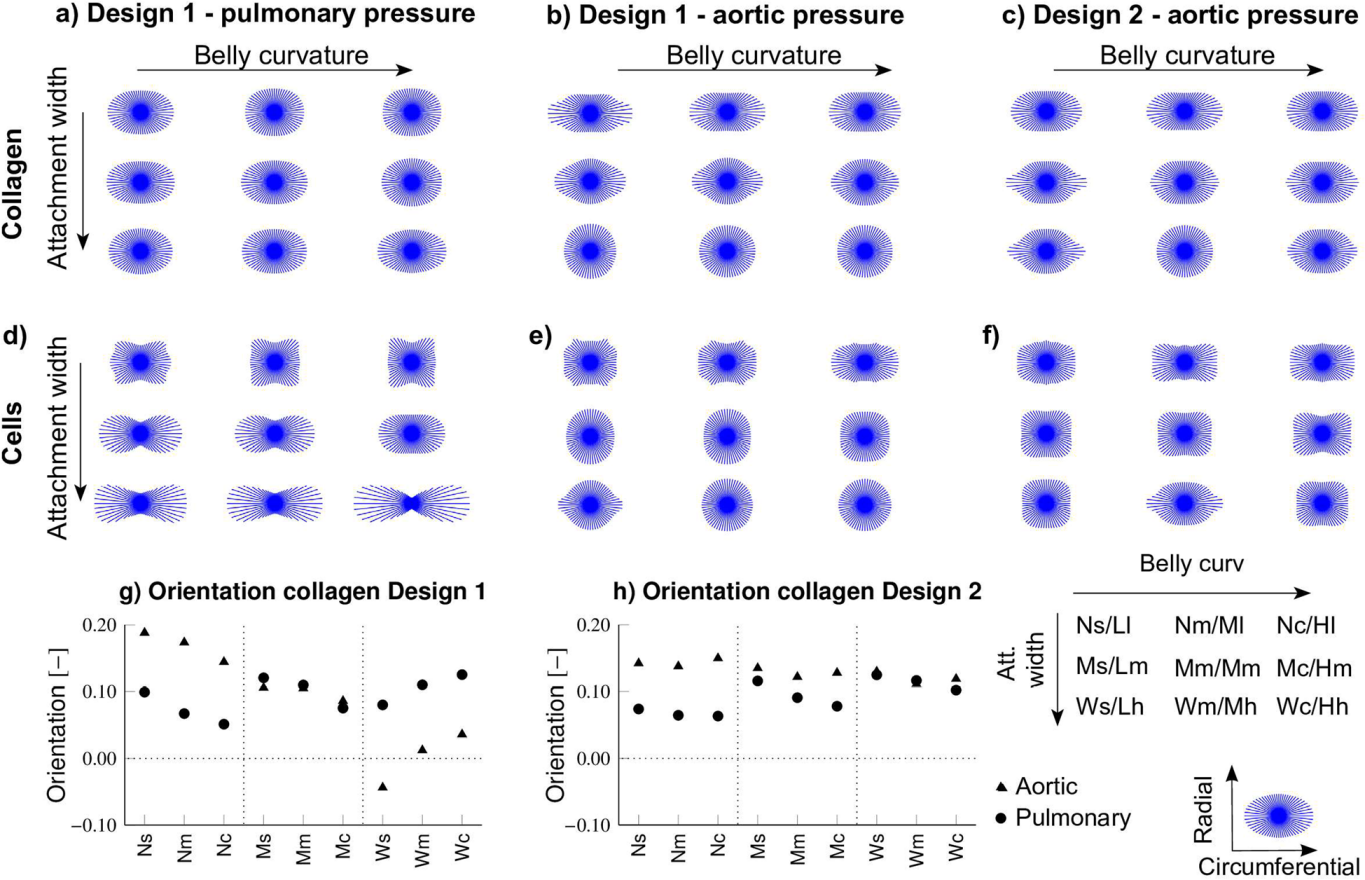
Collagen fiber orientation after remodeling under pulmonary and aortic pressure conditions in Design 1 (**a** and **b**) and aortic conditions in Design 2 (**c**). Cellular stress fiber orientation after remodeling under pulmonary and aortic conditions in Design 1 (**d** and **e**) and aortic conditions in Design 2 (**f**). Orientation parameters of the collagen fiber distribution in pulmonary or aortic pressure conditions in Design 1 (**g**) and Design 2 (**h**)

For collagen fibers, the magnitude of the strain was the dominant factor in their reorganization. Remodeling under pulmonary pressure conditions led to the development of mildly anisotropic collagen fiber distributions with the main fiber angle in the circumferential direction in all geometries of both designs (Fig. 8a, c). This anisotropy emerged through the lower strain-dependent degradation of circumferentially-oriented compared to radially-oriented collagen fibers due to the higher strains in the circumferential direction.

Under aortic conditions, the design played a substantial role in the mechano-regulated evolution of the collagen architecture (Fig. 8g, h). In all geometries of Design 2 and geometries of Design 1 with narrow attachment edges, a highly anisotropic strain is present under aortic pressure (negative strain radially and positive strain circumferentially), resulting in relatively high collagen degradation in the radial direction and minor collagen degradation in the circumferential direction, leading to an anisotropic collagen architecture. However, geometries of Design 1 with wider attachment edges experienced high, but rather isotropic strain, causing low collagen degradation in all directions and, therefore, less anisotropic collagen fiber distributions.

In conditions with low values of absolute strain, such as geometries with wide attachment edges under pulmonary pressure conditions, the retraction was the dominant factor directing stress fiber polymerization. In these geometries, relatively high retraction in the radial direction decreased stress fiber polymerization in this direction compared to the circumferential direction (Sect. 3.3 and Fig. 8d). Since retraction was much larger in TEHVs subjected to pulmonary compared to aortic pressures, the retraction-mediated increase in anisotropy in geometries with wide attachment edges was more prevalent under pulmonary loading conditions.

On the other hand, in conditions with high values of strain, such as aortic pressure conditions, the strain rate was the dominant factor directing stress fiber polymerization in geometries with narrow attachment edges. High absolute strains in the circumferential direction also induced high circumferential strain rates. Since high strain rates decreased stress fiber polymerization, a less circumferentially anisotropic stress fiber polymerization was observed in aortic versus pulmonary pressure conditions in these geometries (Fig. 8e, f).

### Qualitative differences between geometrical designs are preserved with changes in leaflet thickness, coaptation length, and cell contractility

**Table 2:** Variations in parameters to assess their effect on valve insufficiency and stress concentrations

	Profile height	Contractility	Stiffness		Thickness
	*H* [mm]	$\sigma _{{\max }}$ [kPa]	$k_1^{sf}$ [MPa]	$k_2^{sf}$ [-]	*t* [mm]
Baseline	14.3	40 kPa	33.01	11.74	0.6
Adaptation	16.3	20 kPa	36.31	12.91	0.4

**Fig. 9:**
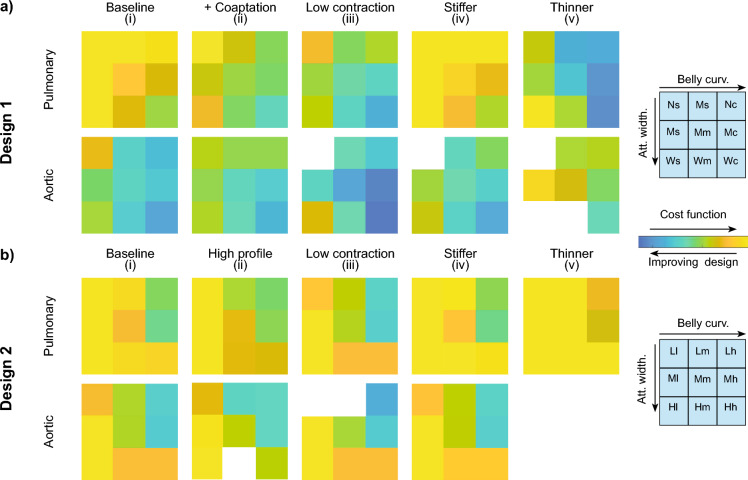
Cost function results of TEHV design variations. Baseline evaluation, as well as evaluations of variations of geometries with additional coaptation, lower cell contractility, stiffer material, and thinner leaflets for Design 1 (**a**) and Design 2 (**b**). The implementation of thin leaflets in aortic conditions led to excessive deformation preventing convergence of the simulations

When evaluating stress concentrations and valve insufficiency, based on the ROA, across various geometries using a cost function, distinct effects of belly curvature and attachment width on overall TEHV function and remodeling were observed (Fig. 9*baseline*). Specifically, increasing the belly curvature improved valve function and remodeling by reducing both stress concentrations and the development of valve insufficiency. Interestingly, the attachment width has opposing effects on remodeling and stress concentrations. Increasing the attachment width reduced the cost value by decreasing the development of valve insufficiency, whereas decreasing the attachment width reduced stress concentrations, especially in geometries of Design 2. Additionally, the smoother leaflets of Design 1 performed better by reducing stress concentrations due to the absence of sharp geometrical transition regions in the leaflets. Overall, these findings indicate that a geometry featuring smooth leaflets, a curved belly profile, and a medium to wide attachment edge profile provide an optimal balance to achieve low stress concentrations and minimized valve insufficiency.

**Fig. 10:**
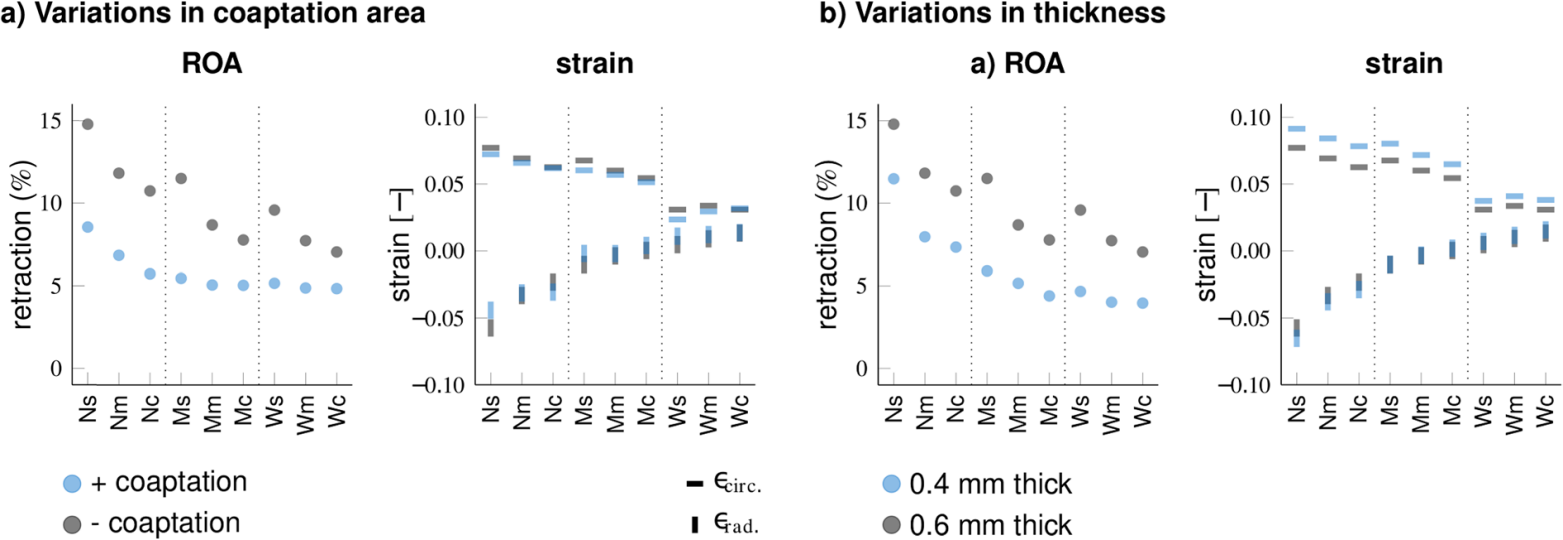
The effect of variations in coaptation (**a**) and leaflet thickness (**b**) on retraction and radial and circumferential strain

Although additional systematic variations of valve designs (Table 2) led to quantitative differences in remodeling and stress concentrations, the overall effects of belly curvature and attachment width on TEHV functionality and remodeling were preserved (Fig. 9). Enhanced coaptation and decreased cell contractility improved valve functionality by reducing valvular insufficiency, with minor effects on strain or stress in the leaflets (Fig. 10a). Lower tissue stiffness or thinner leaflets decreased the retraction seen in pulmonary conditions by increasing the strain (Fig. 10b), but simultaneously increased stress concentrations. Despite these variations, the optimal valve geometry remained consistent across different design modifications. Furthermore, it should be noted that the optimal design could be tailored to specific pressure conditions: thicker or stiffer leaflets for aortic pressure conditions to avoid excessive stresses and strains, and thinner or less stiff leaflets for pulmonary pressure conditions to avoid the development of valvular insufficiency.

## Discussion

In our study, we utilized a bio-inspired computational framework to systematically investigate the impact of geometrical design on mechano-mediated remodeling in TEHVs. Two parameterized valve designs were evaluated under both pulmonary and aortic pressure conditions, focusing on variations in the curvature of the leaflet belly and attachment edge. The distributions of stresses and strains were assessed to predict the risk of fatigue and to analyze the starting point for mechano-mediated remodeling in TEHVs. Additionally, various measures for tissue constituent remodeling and valve functionality were evaluated to enhance our understanding of tissue-level remodeling. Finally, an analysis of geometries with additional coaptation, thicker leaflets, a lower tissue stiffness, and lower cell contractility was conducted to investigate the effects of these variations on valve functionality and stress concentrations.

The current study aimed to develop conservative predictions of the risk of valve insufficiency after remodeling in TEHVs. Therefore, substantially higher contractility and leaflet thickness were adopted compared to the previous studies using in vivo derived data, as these parameters have been shown to increase the predicted retraction (both in Sect. 3.5 and Emmert et al. (2018)). Therefore, the current predictions are considered conservative regarding the development of valve insufficiency, providing a safety margin for the potential future manufacturing of these TEHVs. Furthermore, the higher retraction increased the inter-geometry differences, highlighting small but possibly crucial variations in remodeling across different geometries.

### A TEHV geometry that reduces stress concentrations should be used in order to reduce the risk of fatigue and calcification

Preventing stress concentrations in TEHVs is crucial, not only because they are known to accelerate structural deterioration of materials, but also because they are associated with an increased risk of calcification in heart valves (Gomel et al. 2019; Balachandran et al. 2010; Hasan et al. 2014). In the current study, commissure regions and sharp transition regions in TEHVs were prone to such stress concentrations. This aligns with a study of Travaglino et al. (2020) investigating stress distributions in bioprosthetic heart valve replacements, which also experienced stress concentrations in transition regions from the belly to the coaptation area. Furthermore, increasing attachment width and belly curvature have shown to reduce stress concentrations, which aligned with the previous studies investigating stress concentrations in bioprosthetic heart valves (Travaglino et al. 2020; Abbasi and Azadani 2020). Therefore, smooth leaflets with curved belly profiles and wide attachment edges can minimize stress concentrations and thereby lower the risk of fatigue and calcification in TEHVs.

### A combination of initial valve geometry and pressure conditions should be considered to prevent the development of valve insufficiency

The results indicate that valve geometry and hemodynamic loading conditions substantially affect the mechanical state of TEHVs, which serves as a starting point for mechano-driven remodeling. Specifically, increasing the belly curvature and attachment width increases the strain in radial direction, which, in turn, reduces retraction of the leaflets. In addition to that, aortic pressure conditions resulted in substantially higher deformation compared to pulmonary pressure conditions across all geometries, thereby reducing the compaction of the leaflets. This observation aligns with the previous studies (Loerakker et al. 2016).

Furthermore, our simulations indicated that geometries with longer initial leaflet lengths have a lower risk of developing valve insufficiency. In line with this, the in vivo study of Emmert et al. (2018) has shown that, upon in vivo implantation, TEHVs with curved belly profiles and large coaptation areas shorten in length, but do not develop valve insufficiency because the final leaflet length remains sufficiently long to maintain valve closure.

### The current predictions did not yield a geometry that developed native-like anisotropic collagen and cell alignment

Collagen fibers in native heart valves form thick bundles that are highly circumferentially aligned in a hammock-like structure (van Geemen et al. 2016; Schoen 2018; Misfeld and Sievers 2007; Oomen et al. 2016). However, in vivo remodeling in TEHVs implanted for up to a year in the pulmonary position has shown only mild circumferential collagen alignment (Emmert et al. 2018). This result was also predicted by in silico models of TEHV remodeling using the same computational framework as in our current study (Emmert et al. 2018).

In the present study, we computationally investigated to what extent the resulting collagen organization after remodeling can be improved via manipulating the TEHV geometry. Our simulations suggest that with the current tissue properties, the geometrical variations that we investigated are not able to establish a native-like, circumferential anisotropic collagen fiber organization in TEHVs. Even with circumferential pre-alignment of the collagen fibers before remodeling, no substantial circumferential collagen alignment could be obtained (see Appendix 4). However, it is possible that other mechanisms, not incorporated in the current model, can affect the collagen architecture over longer timeframes. Therefore, long-term in vivo studies are necessary to investigate whether these implants will develop more anisotropic, native-like collagen alignment over extended periods.

However, other types of in situ TEHVs, beyond those explored in this study, may be manufactured to include pre-aligned materials. Examples include techniques such as 3D bioprinting (Zeugolis et al. 2008), anisotropically electrospinning supramolecular elastomeric polymers (Uiterwijk et al. 2020), electrospinning multilayered aligned fibers of electrospun poly(glycerol sebacate) (Masoumi et al. 2014), or using advanced melt electrowriting techniques to develop not aligned and spatially heterogeneous poly($\epsilon$-caprolactone) scaffolds (Saidy et al. 2022). These techniques enable the inclusion of substantially stiffer fibers, which may alter the initial mechanical environment that shapes the remodeling response. However, investigating these TEHVs is outside the scope of this study, as it would require incorporating fibers with distinct material properties into the model, which has not yet been tested or validated.

### A general optimal geometry for TEM-based TEHVs has been identified that may be suitable for both aortic and pulmonary applications

**Fig. 11:**
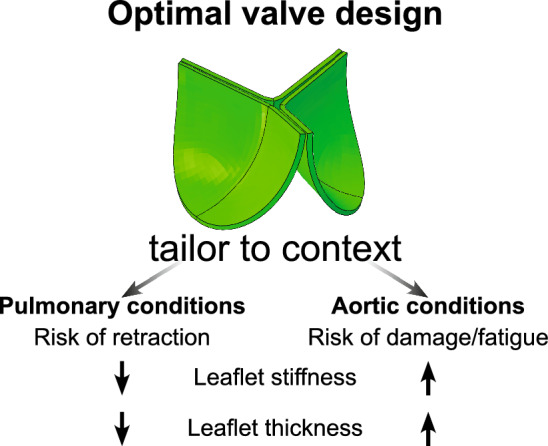
A general optimal valve design has been defined. Tailoring the valve for pulmonary conditions to prevent retraction involves using thinner and softer leaflets. Conversely, tailoring the geometry for aortic pressure conditions to prevent stress concentrations requires thicker and stiffer leaflets

Overall, the design with smooth leaflets, a medium to wide attachment edge width, and a curved belly profile (Fig. 11) has been predicted to be most preferable for both pulmonary and aortic conditions. This design minimizes stress concentrations and reduces the risk of valve insufficiency, as reflected in low cost function results in the analysis in subsection 3.5. Although variations such as thinner leaflets, stiffer collagen properties, larger coaptation area’s, and stronger cellular compaction quantitatively changed the results, qualitative differences in the effect of design features on valve performance were maintained. Further refinement of this TEHV geometry for pulmonary pressure conditions should focus on preventing the development of valve insufficiency by using thinner leaflets, less stiff materials, or longer coaptation lengths. For aortic conditions, on the other hand, the focus should be on reducing stress concentrations with thicker leaflets or stiffer materials.

The novelty of the current study lies in the development of a valve design predicted to maintain long-term functionality under both aortic and pulmonary pressure conditions, unlike prior studies that focused solely on pulmonary valves (Emmert et al. 2018; Sanders et al. 2016). Furthermore, the insights from the optimal design increase confidence in the durability of TEHVs, as they are specifically optimized to minimize stress concentrations, a parameter not previously assessed for TEM-based TEHVs. Additionally, the insights from this study may serve as guidelines for developing TEHVs with modified properties, although verification studies will be necessary to confirm their applicability.

### Limitations

The current study demonstrated how design features affect remodeling in TEHVs under both pulmonary and aortic pressure conditions. Optimizing TEHV design may theoretically benefit from a more generic geometric framework, as demonstrated in several previous studies (Abbasi and Azadani 2020; Haj-Ali et al. 2012; Labrosse et al. 2006; Aggarwal and Sacks 2016; Pan et al. 2024). Additionally, a comprehensive optimization strategy that assesses valve functionality and damage risk could enhance the design process. Because the current framework accounts for the remodeling response, it is computationally expensive, limiting the feasibility of optimization algorithms that require hundreds of simulations.

More generally, this optimized geometry is specific to TEM-based TEHVs with properties within the range tested in this study. Materials with substantial polymeric scaffolds or significantly different material properties are likely to undergo different remodeling processes and may require alternative design features to achieve adaptive remodeling.

Finally, although these results are encouraging, further in vivo studies are necessary to validate the assumptions underlying the current computational framework. While incorporating such studies was beyond the scope of this work, they represent crucial steps toward advancing the models and implementing the optimized design.

## Conclusion

Using a computational framework of mechano-mediated tissue remodeling, we systematically investigated the impact of leaflet design on the remodeling and functionality of tissue-engineered heart valves (TEHVs) under both pulmonary and aortic conditions. Our study demonstrated that hemodynamic conditions, valve geometry, and the material properties of TEHVs substantially affect the remodeling and final valve functionality. In this regard, a common general valve geometry, featuring smooth leaflets, curved belly profiles, and medium to wide attachment edges, suits both pulmonary and aortic conditions. Additionally, it is crucial to tailor the design to prevent excessive retraction in pulmonary conditions, while design adjustments should focus on preventing stress concentrations in aortic conditions. These findings provide valuable guidelines for optimizing TEHV designs to ensure functional remodeling and sustained functionality, paving the way for the development of next-generation TEHVs with improved long-term outcomes.

## Supplementary Information

Below is the link to the electronic supplementary material.

Supplementary file 1 (pdf 3272 KB)

## Data Availability

Computational codes are available at 10.5281/zenodo.13378689, full datasets are available upon request to the corresponding authors.
