# Supplementary material for: Smooth leaflets with curved belly and attachment edge profiles promote adaptive remodeling in tissue-engineered heart valves: an in silico study
Source: Biomech Model Mechanobiol. 2025 Apr 4;24(3):811–28. doi: 10.1007/s10237-025-01937-8 (PMC12162809; doi:10.1007/s10237-025-01937-8)
Supplement: Supplementary file 1 — (pdf 3272 KB) [file 10237_2025_1937_MOESM1_ESM.pdf]

Supplementary information.

1 Design formulation Design 1

Table 3 Parameter values used for design 1. Values $P1_z$, $P2_y$ and $P5_y$ were varied to obtain different valve geometries.

**Attachment curve**	P0	P1	P2
**X coordinate**	-	-	-
**Y coordinate**	0	0	0 - H
**Z coordinate**	0	0-$P2_z$	$P3_z$
	P3		
**X coordinate**	-		
**Y coordinate**	H		
**Z coordinate**	$-(2*\pi/6)*R$		

**Belly curve**	P4	P5	P6
**X coordinate**	-R	0.125	0.125
**Y coordinate**	0	0 - H	$P6_y = P3_x - \lvert\sin(\frac{17}{180})\rvert(\frac{2\pi R}{3}/2)$
**Z coordinate**	-	-	-

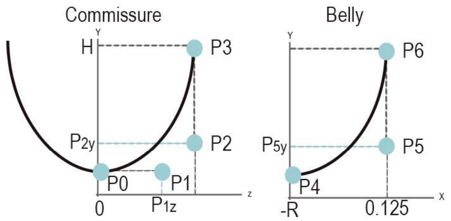

Fig. 12 Essential curves of design 1.

2 Material model

2.1 Relationship between stress and strain of stress fibers

As described in the methods section, for each stress fiber volume fraction the stress in direction i is dependent on the Green Lagrange strain (ϵ^i) and strain rate ($\dot{\epsilon}^i$):

$$\sigma^i_{sf} = \sigma_{max}(f_{\epsilon,a}(\epsilon^i) + f_{\epsilon,p}(\epsilon^i)) \cdot f_{\dot{\epsilon}}(\dot{\epsilon}^i), \quad (17)$$

The active actomyosin contraction is described as:

$$f_{e,a}(\epsilon^i) = \exp\left(-\left(\frac{\epsilon^i}{\epsilon_0}\right)^2\right). \quad (18)$$

Here, parameter ϵ_0 describes the decrease in active contraction with increases in stretch.

Passive strain hardening was described as:

$$f_{e,p}(\epsilon^i) = \begin{cases} (\frac{\epsilon^i}{\epsilon_1})^2, & \epsilon^i \geq 0 \\ 0, & \epsilon^i < 0 \end{cases}, \quad (19)$$

where ϵ_1 describes the passive hardening response of the stress fibers. Furthermore, strain rate influenced stress generation according to:

$$f_{\dot{\epsilon}}(\dot{\epsilon}) = \frac{1}{1 + \frac{2}{\sqrt{5}}}\left(1 + \frac{k_v\dot{\epsilon^i} + 2}{\sqrt{(k_v\dot{\epsilon^i} + 2)^2 + 1}}\right), \quad (20)$$

where parameter k_v resembles the degree of stress reduction in the stress fiber stress with increasing strain rate.

2.2 Stress fiber remodeling

As indicated in the main body of the text, Ristori et al. (2016) et al. developed a method to increase the computational efficiency of the prediction of stress fiber remodeling. Specifically, they showed that the stress fiber volume fraction φ^i_{sf} tends to evolve towards preferred value $\varphi^i_{sf,p}$. They derived that for periodic loading conditions, the stress fiber volume fractions tend towards a function $\tilde{\varphi}^i_{sf}$, which are periodic functions with small oscillations with period T around a constant value:

$$\varphi^i_{sf,p} := \frac{1}{T}\int_0^T \tilde{\varphi}^i_{sf} dt \approx \frac{\bar{a}_i}{\sum_{j=1}^N \frac{\bar{a}_j}{N} + k_d}\phi_a, \quad (21)$$

with

$$\bar{a}_i = \frac{1}{T}\int_0^T \left[k_0^f + \frac{k_1^f \sigma_{max}}{1+2\sqrt{5}} \cdot \left(1 + \frac{k_v \dot{\epsilon} + 2}{\sqrt{(k_v \dot{\epsilon}^i + 2)^2 + 1}}\right) \exp\left(-\left(\frac{\epsilon^i}{\epsilon_0}\right)^2\right)\right] dt. \tag{22}$$

The temporal evolution is subsequently defined by the difference of the current volume fraction and the preferred volume fraction and the time constant τ_s:

$$\frac{d\varphi_{sf}^i}{dt} = \frac{1}{\tau_s}(\varphi_{sf,p}^i - \varphi_{sf}^i). \tag{23}$$

2.3 Collagen remodeling

The total degradation of collagen was constrained by $D_{max} = 1.0$ and $D_{min} = 0.1$ as the maximum and minimum degree of degradation, respectively. It is assumed that collagen is protected from degradation when it is strained according to:

$$\frac{d\varphi_{cf,deg}^i}{dt} = \left(D_{min} + \frac{D_{max} - D_{min}}{1 + 10^{200(\epsilon_e^i - \epsilon_{trans})}}\right)\frac{\varphi_{cf}^i}{\tau_{cf}}, \tag{24}$$

where degradation speed was affected by time constant τ_{cf} and the transition strain $\epsilon_{trans} = 0.12$. Additionally, the elastic Green Lagrange strain ϵ_e^i was the average of the strain in fiber direction i in the unloaded and loaded configuration.

Simultaneously, collagen is produced in an isotropic fashion. Thereby the deposited collagen volume was constrained by the amount of degraded collagen to maintain a constant volume fraction:

$$\frac{d\varphi_{cf,prod}^i}{dt} = \frac{1}{N}\sum_{i=1}^{N} \frac{d\varphi_{cf,deg}^i}{dt}. \tag{25}$$

Ristori, T., Obbink-Huizer, C., Oomens, C.W.J.W.J., Baaijens, F.P.T.P.T., Loerakker, S.: Efficient computational simulation of actin stress fiber remodeling. Computer Methods in Biomechanics and Biomedical Engineering 19(12), 1347–1358 (2016) https://doi.org/10.1080/10255842.2016.1140748 . Publisher: Taylor and Francis Ltd. Accessed 2021-01-19

3 Taguchi analysis of parameter sensitivity

The goal of the study was to investigate the effect of a design parameter on remodeling. Therefore, we analyse three geometries, with changes in belly curvature or attachment edge shape. To evaluate whether the changes in parameter values only lead to qualitative differences in outcome with similar differences between different valve geometries, we evaluated whether the difference between the ROA of a design with a wider attachment edge (ROA_{wc}) and a design with a narrow attachment edge (ROA_{nc}) has the same sign ($\Delta_{attachment} = ROA_{nc} - ROA_{wc}$). Similarly, we assessed whether the difference between the ROA of a design with a curved belly (ROA_{wc}) and a design with a straight belly (ROA_{ws}) has the same sign ($\Delta_{belly} = ROA_{ws} - ROA_{wc}$).

Table 4 Parameter values varied during Taguchi analysis.

Parameter	Baseline value
τ_{sf}	5 min
τ_{λ}	1 h
τ_{cf}	12 h
κ_d^{sf}	$1.0 \cdot 10^{-3}$ s^{-1}
G	$1.0 \cdot 10^{-3}$ MPa
κ_0^{sf}	$1.5 \cdot 10^{-6}$ s^{-1}
ϕ_{cf}	0.5 (-)
ϵ_{trans}	0.12
k_1^{cf}	33.01 kPa
k_2^{cf}	11.74 (-)
σ_{max}	40 kPa

In order to assess the influence of changes in remodeling or material properties on the TEHV remodeling we selected 11 of the most important parameters. First, we assessed the effects of maximum cellular contractility (σ_{max}) as previous studies highlighted its crucial effect on valve remodeling (Loerakker et al., 2016). We also addressed the parameters influencing remodeling speed ($\tau_{sf}, \tau_{\lambda}, \tau_{cf}, k_d$). As previous studies indicated the possible importance of collagen fiber stiffness on remodeling, we included both parameters on collagen and stress fiber stiffness ($k_0^{sf}, k_0^{cf}, k_1^{cf}$), as well as GAG stiffness (G). Furthermore, ϵ_{trans} was assessed as it affects collagen remodeling, and the volume fraction of collagen was adapted. An over view of these variables is provided in Table 4.

We utilized a Taguchi orthogonal array to analyze the influence of these 11 parameters (L12) as described previously (Hisam et al., 2024). All parameters were assessed on values at baseline or 10% higher than their baseline value. For each simulation we

assessed $\Delta_{attachment} = ROA_{nc} - ROA_{wc}$ and $\Delta_{belly} = ROA_{ws} - ROA_{wc}$ to determine if the qualitative effect of changing belly curvature or attachment was maintained with changes in material parameters.

Table 5 Orthogonal array used during Taguchi analysis. Value 1 indicates the use of the baseline parameter value of Table 4, Value 2 indicates the use of a value 10% higher than the baseline value.

**Variable**	**1**	**2**	**3**	**4**	**5**	**6**	**7**	**8**	**9**	**10**	**11**
	τ_{sf}	τ_{λ}	τ_{cf}	κ_d^{sf}	G	κ_0^{sf}	ϕ_{cf}	ϵ_{trans}	k_1^{cf}	k_2^{cf}	σ_{max}
**Run 1**	1	1	1	1	1	1	1	1	1	1	1
**Run 2**	1	1	1	1	1	2	2	2	2	2	2
**Run 3**	1	1	2	2	2	1	1	1	2	2	2
**Run 4**	1	2	1	2	2	1	2	2	1	1	2
**Run 5**	1	2	2	1	2	2	1	2	1	2	1
**Run 6**	1	2	2	2	1	2	2	1	2	1	1
**Run 7**	2	1	2	2	1	1	2	2	1	2	1
**Run 8**	2	1	2	1	2	2	2	1	1	1	2
**Run 9**	2	1	1	2	2	2	1	2	2	1	1
**Run 10**	2	2	2	1	1	1	1	2	2	1	2
**Run 11**	2	2	1	2	1	2	1	1	1	2	2
**Run 12**	2	2	1	1	2	1	2	1	2	2	1

Fig. 13a confirms that a more curved attachment edge consistently leads to reduced retraction, as indicated by a decrease in ROA. Across all 12 parameter combinations (Table 5), the ROA of the design with a wide attachment edge is lower than that of the design with similar belly curvature but a narrow attachment edge, resulting in a positive $\Delta_{attachment}$. Similarly, Fig. 13b confirms that a more curved belly profile consistently leads to reduced retraction, as indicated by a decrease in ROA. Across all 12 parameter combinations (Table 5), the ROA of the design with a curved belly profile is lower than that of the design with a similar attachment edge but a straight belly profile, resulting in a positive Δ_{belly}.

These results indicate that parameter value changes did not qualitatively affect a design parameter's effect on ROA.

Loerakker, S., Ristori, T., Baaijens, F.P.T.: A computational analysis of cell-mediated compaction and collagen remodeling in tissue-engineered heart valves. Journal of the Mechanical Behavior of Biomedical Materials 58, 173–187 (2016) https://doi.org/10.1016/j.jmbbm.2015.10.001 . Accessed 2023-10-10

Hisam, M.W., Dar, A.A., Elrasheed, M.O., Khan, M.S., Gera, R., Azad, I.: The Versatility of the Taguchi Method: Optimizing Experiments Across Diverse Disci- plines. Journal of Statistical Theory and Applications (2024) https://doi.org/10.1007/s44199-024-00093-9 . Accessed 2024-11-14

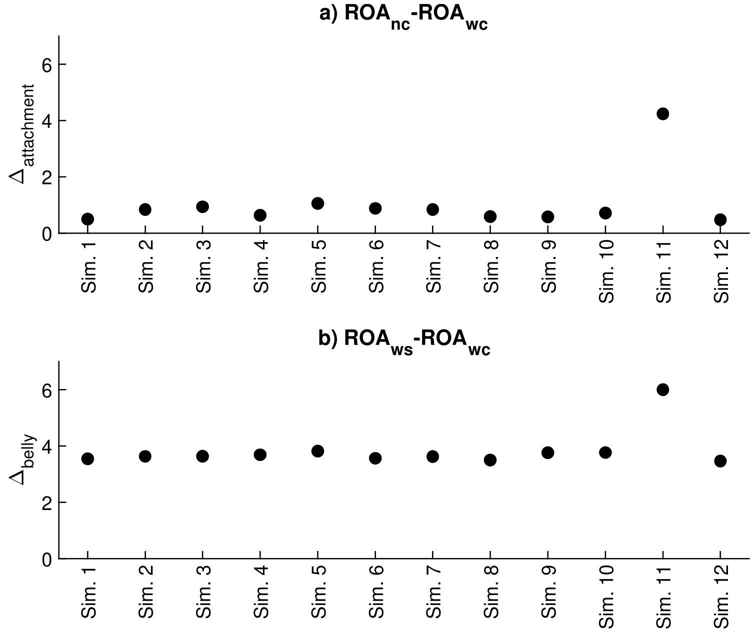

Fig. 13 Taguchi analysis of parameter sensitivity of the model. Simulations used different combinations of parameter values based on Table 5. Subsequently, the difference in the percentage ROA was calculated. a) Difference in ROA for designs with similar belly curvature but different attachment edges. b) Difference in ROA for designs with similar attachment edges but different belly curvatures.

4 Initial anisotropic collagen fiber distribution

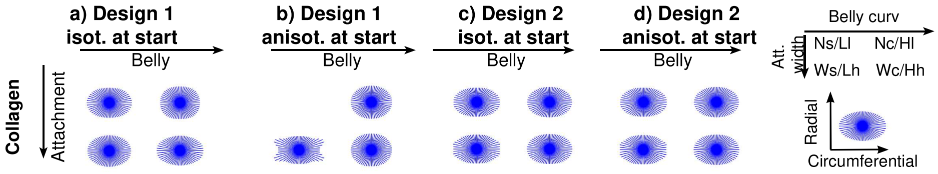

Fig. 14 Collagen fiber alignment in TEHVs after remodeling under pulmonary pressure conditions. a) starting with isotropic collagen fiber alignment in geometries of Design 1 (repetition 8a), b) starting with anisotropic collagen fiber alignment in geometries of Design 1, c) starting with isotropic collagen fiber alignment in geometries of Design 2, d) starting with anisotropic collagen fiber alignment in geometries of Design 2

Since no reorganization towards native-like circumferential collagen fibers was observed with initially isotropic collagen, we investigated whether more circumferentially anisotropic, native-like collagen fiber distributions could be achieved using materials with strongly anisotropic collagen orientation prior to remodeling. To test this, we simulated valves with pronounced anisotropic collagen fiber orientation, with the main fiber angle aligned circumferentially prior to remodeling. Since remodeling in valves under pulmonary pressure resulted in more circumferentially anisotropic collagen, we tested merely remodeling under pulmonary pressure conditions. Four geometries for each parametrized design were tested, with the most curved and straight belly profiles, as well as the most wide (u-shaped) or narrow (v-shaped) attachment edges. One simulation did not converge, due to buckling of the leaflet, which induced leaflet prolapse.

After remodeling, a similar final collagen fiber distribution was observed in valves with identical geometry but differing initial collagen fiber orientations. Hence, despite the adapted initial fiber orientation, no native-like fiber orientation was present after remodeling. This suggests that for final collagen fiber reorientation the influence of hemodynamics overrules initial fiber orientation in TEHVs made from TEMs.
